# Abiotic-biotic characterization of Pt/Ir microelectrode arrays in chronic implants

**DOI:** 10.3389/fneng.2014.00002

**Published:** 2014-02-04

**Authors:** Abhishek Prasad, Qing-Shan Xue, Robert Dieme, Viswanath Sankar, Roxanne C. Mayrand, Toshikazu Nishida, Wolfgang J. Streit, Justin C. Sanchez

**Affiliations:** ^1^Department of Biomedical Engineering, University of MiamiCoral Gables, FL, USA; ^2^Department of Neuroscience, University of FloridaGainesville, FL, USA; ^3^Department of Electrical and Computer Engineering, University of FloridaGainesville, FL, USA; ^4^Department of Neuroscience, University of MiamiCoral Gables, FL, USA; ^5^Miami Project to Cure Paralysis, University of MiamiMiami, FL, USA

**Keywords:** Pt/Ir microelectrodes, neural interface, blood brain barrier (BBB), neuroinflammation, abiotic, biotic, floating microelectrode arrays (FMA), impedance

## Abstract

Pt/Ir electrodes have been extensively used in neurophysiology research in recent years as they provide a more inert recording surface as compared to tungsten or stainless steel. While floating microelectrode arrays (FMA) consisting of Pt/Ir electrodes are an option for neuroprosthetic applications, long-term *in vivo* functional performance characterization of these FMAs is lacking. In this study, we have performed comprehensive abiotic-biotic characterization of Pt/Ir arrays in 12 rats with implant periods ranging from 1 week up to 6 months. Each of the FMAs consisted of 16-channel, 1.5 mm long, and 75 μm diameter microwires with tapered tips that were implanted into the somatosensory cortex. Abiotic characterization included (1) pre-implant and post-explant scanning electron microscopy (SEM) to study recording site changes, insulation delamination and cracking, and (2) chronic *in vivo* electrode impedance spectroscopy. Biotic characterization included study of microglial responses using a panel of antibodies, such as Iba1, ED1, and anti-ferritin, the latter being indicative of blood-brain barrier (BBB) disruption. Significant structural variation was observed pre-implantation among the arrays in the form of irregular insulation, cracks in insulation/recording surface, and insulation delamination. We observed delamination and cracking of insulation in almost all electrodes post-implantation. These changes altered the electrochemical surface area of the electrodes and resulted in declining impedance over the long-term due to formation of electrical leakage pathways. In general, the decline in impedance corresponded with poor electrode functional performance, which was quantified via electrode yield. Our abiotic results suggest that manufacturing variability and insulation material as an important factor contributing to electrode failure. Biotic results show that electrode performance was not correlated with microglial activation (neuroinflammation) as we were able to observe poor performance in the absence of neuroinflammation, as well as good performance in the presence of neuroinflammation. One biotic change that correlated well with poor electrode performance was intraparenchymal bleeding, which was evident macroscopically in some rats and presented microscopically by intense ferritin immunoreactivity in microglia/macrophages. Thus, we currently consider intraparenchymal bleeding, suboptimal electrode fabrication, and insulation delamination as the major factors contributing toward electrode failure.

## Introduction

The development of clinically viable microelectrode arrays for humans has produced multiple engineering design and neurophysiological requirements that are believed to be necessary for facilitating high performance (Jackson and Zimmermann, 2012). These include the ability to target and access the activity in ensembles of neurons located in cortical and deep brain structures for treating a variety of neurological problems including paralysis (Ethier et al., 2012; Hochberg et al., 2012; Collinger et al., 2013), stroke (Iosa et al., 2012), movement disorders (Andrews, 2010; Rouse et al., 2011), epilepsy (Morrell, 2011; Truccolo et al., 2011), and neuropsychiatric disorders (Maling et al., 2012). Not only is short-term access of these neurons important but also the microelectrode arrays should be able to sense and stimulate targeted neurons reliably, which could be of the order of tens of years. From a materials science perspective, there are differences in the design considerations between sensing and stimulation but in general, it is desirable to have stable electrodes that do not corrode (Sanchez et al., 2006; Prasad et al., 2012), produce stable impedance (Ward et al., 2009; Prasad and Sanchez, 2012), and are robust after repeated delivery of current through them (Johnson et al., 2005; Otto et al., 2005, 2006; Koivuniemi and Otto, 2011; Lempka et al., 2011). A variety of materials including tungsten, platinum, platinum-iridium, and iridium oxide coatings have been used to improve the reliability of recordings (Geddes and Roeder, 2003; Cogan, 2008; Ward et al., 2009). Closely coupled to the abiotic materials aspects are the biotic responses to chronically implanted microelectrode arrays. The biotic effects include the disruption of the blood-brain barrier (BBB) (Prasad et al., 2011; Karumbaiah et al., 2013; Saxena et al., 2013), tissue inflammatory response involving astroglial and microglial reactions occurring at/around the implanted site, and macrophage recruitment to the implanted site (Schmidt et al., 1976; Stensaas and Stensaas, 1978; Edell et al., 1992; Kam et al., 1999; Turner et al., 1999; Szarowski et al., 2003; Biran et al., 2005; Lee et al., 2005; Polikov et al., 2005; Biran et al., 2007; McConnell et al., 2009b; Winslow and Tresco, 2010; Thelin et al., 2011; Prasad et al., 2012). The abiotic effects include the changes occurring at the electrode recording sites such as corrosion and insulation delamination and cracking that alters the electrochemical properties of the electrode recording surface area (Geddes and Roeder, 2003; Patrick et al., 2011; Prasad and Sanchez, 2012; Prasad et al., 2012; Streit et al., 2012; Kane et al., 2013). Both these effects are dynamic in nature, occur concurrently, and cannot be isolated from one another. Therefore, high-performance arrays should produce minimal tissue reactivity including disruption of the blood-brain-barrier (Prasad et al., 2012), glial response (Frampton et al., 2010; Winslow and Tresco, 2010; Prasad et al., 2012), and neuronal damage (McConnell et al., 2009b). Finding the optimal balance of all of these design considerations is challenging and various electrode tip geometries (Andrei et al., 2012) and multiple array solutions have been proposed including microwire arrays, floating planar silicon arrays, floating 2-D silicon arrays, and floating microwire arrays (Drake et al., 1988; Rousche and Normann, 1998; Williams et al., 1999a,b; Rousche et al., 2001; Csicsvari et al., 2003; Kipke et al., 2003; Nicolelis et al., 2003; Vetter et al., 2004; Patrick et al., 2006; Jackson and Fetz, 2007; Musallam et al., 2007; Kozai et al., 2012a; Kim et al., 2013; Richter et al., 2013). Unique among these available solutions is the “floating” aspect of the array design. It is believed that floating arrays can mitigate many of the challenges of chronic implantation as they have been shown to cause reduced lesions due to electrode array micro-movements in the brain tissue (Biran et al., 2007). Systematic and quantitative study of each of these array types is necessary to prove their benefits and weaknesses in the context of electrode performance and failure.

In this work, floating microelectrode arrays (FMA) fabricated by Microprobes (Gaithersburg, MD) are studied. These electrodes are made of Pt/Ir which provide a more stable and inert electrode-recording site that is less prone to corrosion and surface changes compared to other metals such as tungsten and stainless steel (Geddes and Roeder, 2003; Cogan, 2008; Patrick et al., 2011; Prasad et al., 2012; Kane et al., 2013). In addition, they are laser cut with a conical tip to reduce the trauma during insertion. Unlike the Utah array, which is the only other commercial design that is similar, the FMA can be custom cut for both deep brain and cortical targets. The arrays are fabricated on a planar substrate and connected via a thin flexible cable, which allows the array to float with the brain. While FMAs have been used in a variety of cortical and spinal applications for basic neurophysiological and neuroprosthetic applications, they have not undergone comprehensive characterization to quantify their electrophysiological properties over long-durations (weeks to months) *in vivo*. We have recently developed an *in vivo* testbed (Prasad and Sanchez, 2012; Prasad et al., 2012; Streit et al., 2012) to quantify the failure mechanisms of microelectrodes and have shown that there are a variety of biotic and abiotic aspects that contribute to electrode performance. Since microelectrode array design is so diverse among the available options (Utah, FMA, microwires, Michigan), each design needs to be evaluated independently using a common set of relevant metrics. Toward developing a comprehensive characterization of FMA performance and failure, this study performs an *in vivo* long-term abiotic and biotic analysis with an aim to build a quality control system for microelectrodes. FMAs were implanted into the rat somatosensory cortex for implant duration ranging from 7 days up to 6 months. Abiotic analysis included (1) pre-implant and post-explant SEM imaging for qualitative comparison of the changes that occur at the electrode recording surface, and (2) chronic *in vivo* impedance spectroscopy for studying the changes in the electrode impedance chronically. Biotic analysis was assessed using microglial activation and degeneration and BBB disruption. Chronic electrode performance was quantified via array yield. The array yield, specified by the number of recording sites producing single neuron recordings, provides insight to the overall health of the array. High-performance electrode arrays produce single neuron recordings from a maximal number of recording sites. Likewise, failing electrode arrays produce low yields. It has been shown that impedance spectroscopy provides insight to the fundamental aspects of biotic and abiotic interactions that affect array performance (Grill and Mortimer, 1994; Merrill and Tresco, 2005; Ludwig et al., 2006, 2011; Williams et al., 2007; McConnell et al., 2009a; Karumbaiah et al., 2013). Combining standard electrophysiological measurements with abiotic and biotic analysis can produce comprehensive insight to electrode failure.

## Methods

All procedures were approved by the University of Miami Institutional Animal Care and Use Committee (IACUC). Twelve age-matched adult male Sprague–Dawley rats, all weighing between 300 and 350 grams (at implant time) were used in this study. Animals were divided into short-term implant group (≤2 weeks) (Turner et al., 1999; Winslow and Tresco, 2010) and chronic groups (≤6 months) (Williams et al., 1999b; Polikov et al., 2005; Winslow and Tresco, 2010). In this study, 3 animals were implanted for up to 7 days, 2 for 15 days, 1 for 3 weeks, 3 for up to 3 months, and 3 for 6 months, respectively. The 7-days and 15-days animals were classified in the short-term implant group whereas the remaining animals were classified in the chronic group. All animals were euthanized at the end of their prospective study period intentionally and the brain tissue was harvested for histological analysis. Table 1 summarizes the pre-implant and post-explant SEM observations, functional performance, and histopathological summary for all 12 animals included in the study.

**Table 1:** **Summary of all animals used in this study**.

**Animal groups**	**Rat no**.	**Days post-implant**	**Scanning electron microscopy (SEM) imaging**		**Electrophysiology**			**Histopathology**	
			**Pre-implant**	**Post-explant**	**Impedance (MΩ) on day 1**	**Impedance (MΩ) at euthanasia**	**Average array array yield (%)**	**Activated microglia (Yes/No)**	**Degenerated microglia (Yes/No)**
Short-term implants(≤15 days)	F11	15	Non-uniform insulation, cracks in insulation near recording tips	Bent tips, insulation delamination and cracks in insulation	0.45 ± 0.14	0.689 ± 0.17	39.3 ± 32.8	Y	Y
	F12	7	Crack in no. 1, some wires not straight, non-uniform insulation along wires	Cracks in insulation, insulation delamination, recessed insulation	0.37 ± 0.12	0.504 ± 0.29	65 ± 28.1	Y	Y
	F13	15	Electrode tip not straight for no. 13, cracks along wire shank, some wires not straight	Insulation delamination in some electrodes, cracks in insulation	0.41 ± 0.12	1.27 ± 0.43	78.1 ± 19.6	Y	Y
	F14	7	Electrode tip not straight for no. 16, cracks in insulation along shank	Broken tip, insulation delamination	0.51 ± 0.68	0.494 ± 0.23	33.3 ± 20	Y	Y
	F15	6	No. 2 crack on recording surface, cracks in insulation along shanks, no. 8 wire tip not straight	Electrode tip not straight, some deinsulation	0.4 ± 0.11	0.593 ± 0.22	81.2 ± 22.2	Y	Y
Chronic implants (≤6 months)	F9	71	Majorly flawed: cracks in insulation at recording tips for most electrodes, non-uniform insulation	Cracks in insulation, insulation delamination, recessed insulation	1.44 ± 0.59	0.947 ± 0.26	16.8 ± 22.4	Y	Y
	F10	21	Cracks on electrode shanks, cracks in insulation at recording tips	Cracks, delamination, insulation damage	0.47 ± 0.14	0.594 ± 0.19	4.8 ± 13.7	Y	Y
	F2	180	Non-uniform insulation on all wires, some wires not straight	Some bent tips, two electrodes have cracks in insulation	0.61 ± 0.65	1.96 ± 0.71	74.3 ± 13.9	Y	Y
	F4	180	Irregular insulation, some wires not straight, tip missing for no. 6, bent recording tip no. 9	Bent tips, insulation delamination	0.397 ± 0.134	1.43 ± 0.49	40.5 ± 22.5	N	N
	F6	180	Bent tips for nos. 3, 4, 5, 9, and 13, some wires not straight, cracks in insulation	Insulation delamination on some wires, bent tips	0.43 ± 0.2	0.7 ± 0.3	35.1 ± 19.6	N	N
	F7	90	Moderately flawed: bent tip no. 16, non-uniform insulation at recording tips, crack in insulation at tips	Insulation delamination in some electrodes, cracks, bent tips	0.59 ± 0.7	1.47 ± 0.81	12 ± 8.6	N	Y
	F8	91	Majorly flawed: crack in recording tip no. 5, cracks in insulation of 8 electrodes, some wires not straight	Insulation delamination	1.08 ± 0.24	2.14 ± 0.43	19.5 ± 21.4	Y	Y

Animals are divided into short-term implants (≤2 week) and long-term implants (≤6 months). The table provides summarizes general abiotic and biotic observations.

### Electrode

**Figure 1:**
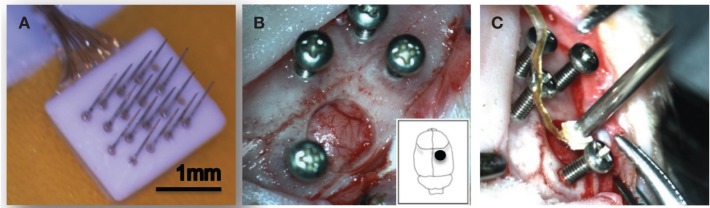
**Experimental setup**. A 16-channel Pt/Ir floating microelectrode array (FMA) consisting of 1.5 mm long and 75 μm diameter with tapered microwires tips was used for all implants **(A)**. Surgical implantation procedure in the rat somatosensory cortex is shown **(B,C)**.

We characterized 16-channel FMA (MicroProbes, Inc, MD) for our implants in all animals (Musallam et al., 2007; Mollazadeh et al., 2011). The microelectrode array consisted of 16 microwires [Pt/Ir (70/30%) 75 μm diameter at the base which tapered to a fine tip] attached to a ceramic substrate (Figure 1A). Each of the microwires were 1.5 mm long and separated by 400 μm. The microwires were insulated with parylene-C, the thickness of which was 3 μm along the wire shaft and the microwires were laser cut at the tip, which produced a conical recording surface. The microwires were soldered to gold wires, which were bundled together and soldered to a 16-channel Omnetics connector that was fixed on the skull. The wire bundle was coated with medical grade silicone to provide insulation.

### Scanning electron microscopy (SEM) imaging

In order to evaluate the structural changes in chronically implanted microelectrode arrays, all arrays were imaged before implantation and after explantation using a variable pressure scanning electron microscope (Hitachi S-3000N VP-SEM). The environmental mode in the VP-SEM was chosen for imaging the arrays to enable direct placement of the samples into the SEM chamber without the use of carbon or conductive coating. This is very critical especially for pristine electrodes as they will be implanted into an animal after the SEM imaging procedure. Such a method was used so as to cause no damage to the arrays from the imaging process for both pre-implant as well as explanted arrays. When the pre-implant images were taken, electrode arrays were handled with care to prevent any damage to the microwires. The post-explant images were taken on the electrode arrays extracted from the implanted tissue. The post-explant arrays were carefully placed on a holder positioned on to the SEM stage. The following parameters were used to take the pre- and post-implant images: (1) environmental secondary electron detector (ESED) mode with an acceleration potential of 12 kV, (2) working distance range was set between 18 and 40 mm, and (3) magnification was varied according to need and the minimum magnification was set at 20X.

### Impedance testing procedure

Electrode impedance spectroscopy was performed using NanoZ (TDT, Alachua, FL) using procedures described in detail elsewhere (Prasad and Sanchez, 2012). Briefly, NanoZ measures impedance by applying a small sinusoidal voltage of 4 mVpp at specific frequencies of 1 Hz, 2 Hz, 5 Hz, 10 Hz, 20 Hz, 50 Hz, 100 Hz, 200 Hz, 500 Hz, 1 kHz, and 2 kHz, respectively. The small amount of voltage applied by NanoZ causes a maximum test current of 1.4 nA RMS to flow through the measuring circuit. The impedance measurement protocol was approved by the electrode manufacturer. We combined repeated impedance measurements following the above protocol with scanning electron microscopy (SEM) imaging to verify that electrode recording surfaces were not altered due to the measurement process itself. Impedance spectroscopy *in vitro* was performed for electrode arrays with respect to a low impedance stainless steel reference wire prior to implantation in 0.9% phosphate buffered saline (PBS) for obtaining baseline values for each array. The two-electrode method has been deemed suitable for measuring microelectrode impedance owing to the large difference in impedance between the electrode being tested and the distant low impedance reference wire (Brett and Brett, 1993; Geddes, 1997; Williams et al., 2007). *In vivo* measurements were made with respect to the same stainless steel wire tied to the skull screw while the animals were lightly anesthetized during impedance measurements. *In vivo*, impedance was measured 3–4 times a week before electrophysiological recordings. In our testing, the NanoZ calculated the impedance values for each electrode by repeating the process 20 times and reported the average of those values to reduce errors due to measurement.

### Surgical procedure

Electrodes were individually sterilized using ethylene oxide gas prior to implantation and sterile surgical techniques were followed for all implant surgeries. Animals were anesthetized with an induction of isoflurane (4%) and O_2_ (2 L/min) and deep anesthesia level was maintained for the entire duration of the surgical procedure using isoflurane (1.5%) and 1 L/min O_2_. Xylazine (5 mg/kg, subcutaneous) was used to maintain an even plane of anesthesia and as a muscle relaxant. Upon fixing the animal's head in a stereotaxic frame (Kopf Instruments, Tujunga, CA) to stabilize the skull, a midline incision was made on the skin from between the eyes up to the ears and the periosteum was scraped from on top of the skull. The skull surface was cleaned and the bregma and lambda landmarks were identified. Four stainless steel screws were manually drilled into the skull. Using stereotaxic coordinates, the craniotomy location (1 mm lateral and 1.8 mm caudal to the bregma) was marked which correspond to the somatosensory cortex. A 5 mm diameter circular craniotomy was drilled at the above marked location (Figure 1B) and the dura was then cut using microscissors to expose the cortical surface. The setup shown in Figure 1C was used to hold the electrode connector and position the electrode array using a vacuum pump wand. The electrode array was slowly (~0.1 mm/min) lowered into the cortex using the micropositioner (Kopf Instruments, Tujunga, CA). Slow speed of insertion was used for these FMA as recommended by the manufacturer and similar to studies that have shown success in recording single neurons with a slow insertion speed for microwire arrays (Nicolelis, 1999). A small piece of gelfoam soaked in sterile saline was used to cover the craniotomy and dental acrylic (A-M Systems, Carlsborg, CA) was placed on and around the craniotomy. Extreme care was taken such that none of the acrylic entered the craniotomy. The ground and reference wires were tied to the skull screw and the entire area was filled with acrylic. Animals were placed under supervision in a recovery box kept on a heating pad post-surgery and housed individually upon recovery. Bupivacaine was used around the wound site after the surgery and Carprofen (5 mg/kg, subcutaneous) was used as the analgesic. No antibiotics or any other drugs were used in this study.

### Recording procedure

Animals were recorded 3–4 times each week. Electrode impedance spectroscopy was performed prior to each recording session. The animal was connected to a preamplifier that was optically isolated from a real-time digital signal processor (RZ2, TDT, FL). A custom made program (RPvDx, TDT, FL) was used to acquire neural signals sampled at 24414.06 Hz. Signals were band-pass filtered between 0.5 and 6 kHz and online spike sorting based on boxes and thresholding as determined by the experimenter was used to isolate neuronal waveforms. Offline spike sorting was then performed to verify isolated single units and remove other artifacts. Waveforms were classified as single units based upon the repeatability of waveform shape and peak-to-peak amplitude with respect to the background noise (Suner et al., 2005). Electrophysiological recordings were quantified via array yield, which was defined as the percentage of electrodes within an array that were able to isolate at least one neuron during a recording session. Array yield was used as a functional measure for electrode assessment. An electrode was said to isolate a neuron (single unit) when at least 100 reproducible biphasic waveforms could be collected during a session.

**Figure 2:**
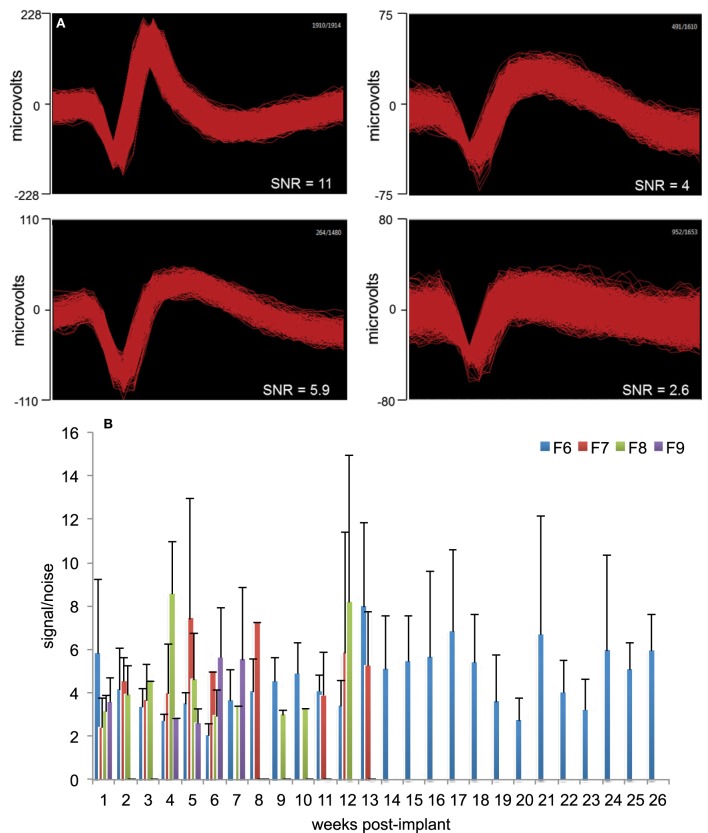
**Signal quality and SNR. (A)** Representative offline sorted waveforms isolated from individual electrodes to depict signal quality. High (>5), medium (3–5), and low SNR (<3) waveforms are shown. In general, SNR values were in the range 3–6. Waveforms are shown within a 1.2 ms time-window. **(B)** Weekly signal-to-noise levels for animals F6, F7, F8, and F9 are shown. SNR values were comparable to those reported earlier by other studies. SNR values were not dependent upon implant durations or daily changes in impedance values.

Signal-to-Noise ratio (SNR) was calculated using the formula: $SNR=\frac{V}{2*\sigma }$, where *V* is the peak-to-peak amplitude of the mean waveform and σ is the standard deviation of the noise. These methods have been described in detail elsewhere (Suner et al., 2005). Figure 2A provides a general measure of the signal quality where offline sorted waveforms from four isolated neurons is shown with SNR value calculated for each neuron. The neuron waveforms shown are for animal F6 and are from different electrodes and different sessions. All waveforms were collected and shown within a 1.2 ms time-window. Weekly signal-to-noise levels are shown for four animals (F6–9) during their respective implant durations in Figure 2B. In general, SNR values ranged between 3 and 6 and were comparable to those reported earlier by other studies (Suner et al., 2005; Ward et al., 2009). We found that SNR values were not dependent upon implant durations or daily changes in impedance values and also not correlated to the electrode performance.

### Immunohistochemical procedures

Immunohistochemical procedures have been described in detail elsewhere (Prasad et al., 2012). Briefly, microglial cells in brain sections were examined immunohistochemically using the rabbit polyclonal primary antibody Iba1 (Wako, 019–19741, diluted at 1:800). Phagocytic microglia were identified using ED1 antibody (mouse anti-rat monocytes/macrophages (CD68), Chemicon; MAB1435, diluted at 1:300). Immunolabeling for the iron storage protein was performed using antiferritin polyclonal antibody (rabbit anti-horse spleen ferritin, Sigma, F6136, diluted at 1:800). Ferritin, an iron storage protein that becomes expressed in some microglia in the human brain are often dystrophic rather than activated (Dijkstra et al., 1985). Ferritin staining of microglia seems to be induced under certain injury/disease conditions when the blood–brain barrier is disturbed and there is a need for iron sequestration. The ferritin antibody also binds to oligodendrocytes in the normal uninjured central nervous system (Lopes et al., 2008). Quantitative analysis of antigen expression was conducted on images using Image J program (NIH, Bethesda, MD). The total positive area of an antigen expression per unit section area (i.e., per microscopic field ×20; total area size 267320.12 μm^2^) were scored in an image captured from each side of rat brain by using Image J program. The mean values and standard deviation of the counts were computed. An intact microglial cell was defined as a cell with a cell body area larger than 30 μm^2^ and less than 300 μm^2^ for Iba1 and ferritin labeling. The ED1 positive particle was defined as areas from 1 to 10 μm^2^. The difference between the electrode implant side and the contralateral side was interpreted to reflect an approximation of microglial cell function.

## Results

### SEM imaging observations

**Figure 3:**
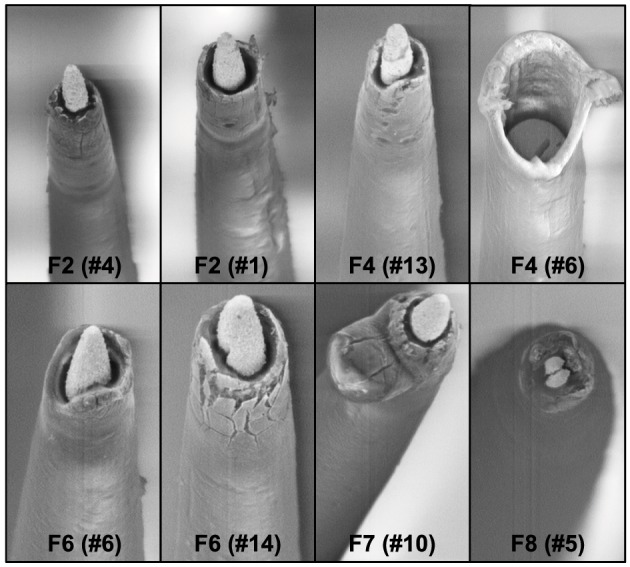
**Manufacturing variability**. We observed large variation between electrodes within the same Pt/Ir array. Shown here are 8 wires from four arrays pre-implant with imperfections as a result of manufacturing process. One of the wires is missing the recording tip whereas others have cracks in the insulation/recording tip, and uneven insulation at the interface of the recording surface/insulation.

**Figure 4:**
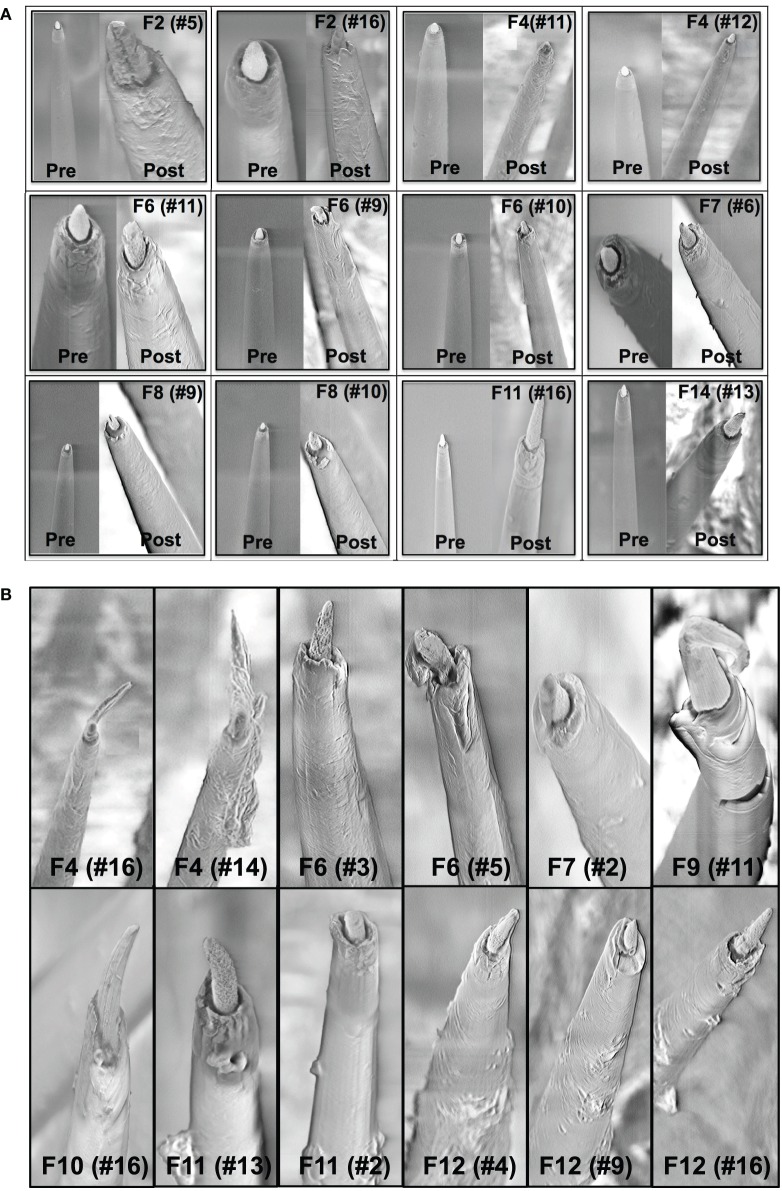
**(A)** Pre-implant and post-explant SEM. Pre-implant and post-explant SEM images to show minimal to moderate changes at the recording site structure for the longest term animals. However, recording site changes occur as a result of corrosion of the recording surface for long-term implants. Shown here are three 6-month implants (F2, F4, F6), two 3-month animals (F7, F8), one 15-day animal (F11), and one 7-day animal (F14), respectively. A crack at the recording site can be observed to be developing in the electrode F2 (no. 5). **(B)** Insulation deterioration post-explant SEM. Post-explant SEM of individual microwire to indicate deterioration in electrode insulation for parylene-C coated Pt/Ir microwires. The deterioration occurs in the form of delamination and cracks. While the insulation deterioration varies among microwires even with the same array, we observed it to be present in all the wires across animals for all implant durations (7 days–6 months).

**Figure 5:**
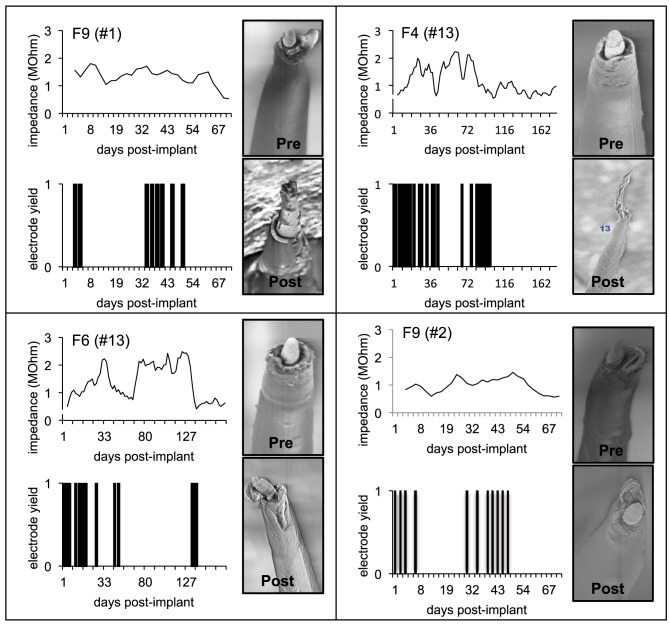
**Effect of insulation damage**. Pre-implant and post-explant SEM images are shown for four Pt/Ir electrodes in three chronic animals that undergoes delamination during their respective implant durations. Insulation delamination results in a decline in 1 KHz electrode impedance combined with poor functional performance (low yield). Functional performance is characterized by electrode yield on each of the recording days. A zero yield suggests that the respective electrode was not able to record a single unit whereas a 1 suggests that the electrode was able to record a single unit.

A comparison of the pre-implant and post-explant SEM images of the electrode arrays was made to analyze qualitatively the changes at the electrode recording surface morphology for all animals. All electrodes prior to implantation were imaged to observe any pre-implant defects in the electrodes due to the manufacturing process. We observed several defects occurring as a result of manufacturing most commonly present in the recording surfaces and the interface between the recording surface and the insulation. The manufacturing defects most commonly present were cracks in the insulation material, non-uniform insulation, bent and broken recording tips, cracks on the recording tips, and non-uniform deinsulation (Figure 3). Figure 3 shows examples of electrodes with pre-implant manufacturing imperfections for 4 of the longest term animals (3–6 months implant period). These images were taken prior to electrode implants. Note that not all the electrodes within an array had issues but approximately 25% or more electrodes tend to have one or the other issue as described above. Thus, there was a large variation between electrodes within an array with some electrodes with no issues whereas others with pre-implant imperfections. Pre-implant and post-explant SEM images were taken for qualitative comparison of the changes that occur at the electrode recording surface and insulation material. Figure 4A shows such changes occurring after chronic implantation in animals where the electrodes underwent moderate to minimal changes at the recording surfaces. We observed that Pt/Ir undergoes little corrosion for implants up to 6 months. However, there was a great variability among individual electrodes even within the same array with regard to corrosion and/or insulation deterioration. While electrode corrosion did not appear to be a significant problem with Pt/Ir, we observed severe delamination and cracking of insulation material. Gross morphological changes occurred at the recording surface in the form of bent tips, cracks appearing in the insulation material, and insulation delamination. Figure 4B show 12 representative electrodes from 7 long-term animals (3–6 months) where severe damage to the insulation material was observed. Insulation damage and delamination resulted in a decrease in electrode impedance during the chronic lifetime and affected functional performance (Figure 5). Figure 5 shows such an example for 4 electrodes from 3 long-term animals (F4 and F6: 6 months; F9: 71 days) where electrode impedance declined during the latter part of the implant period. Interestingly, as the electrode was undergoing these morphological changes, the functional performance (electrode yield) became poor.

### Impedance and electrophysiological observations

**Figure 6:**
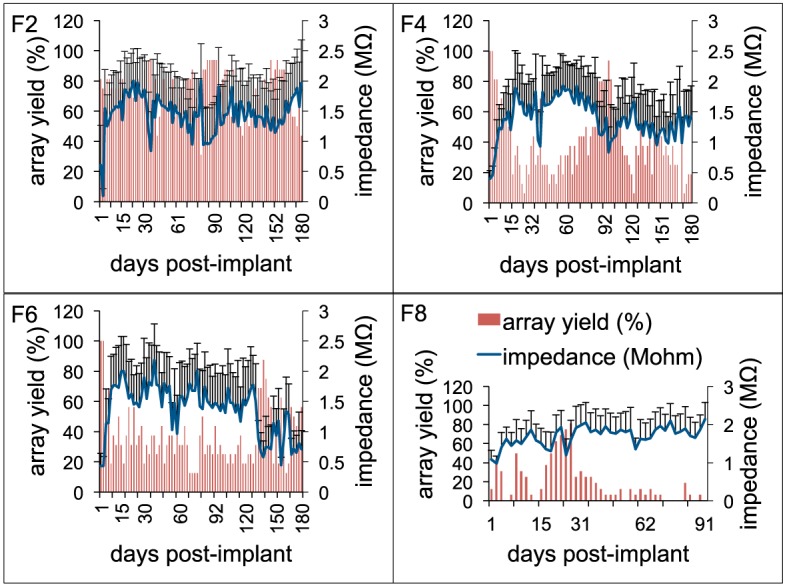
**Electrode impedance and electrophysiology trends**. Daily array yield (red) and average 1 KHz impedance trends (blue) for four long-term animals (F2, F4, F6: 6-months; F8: 3-months) to show the large daily variations in electrode impedance that result in low array yield. Array yield was defined as the percentage of electrodes out of 16 electrodes in an array that was able to isolate at least a single neuron during a recording session. Error bars depict standard deviations among the impedance values recorded from 16 electrodes during a recording session.

To study general trends in impedance magnitude, 1 kHz impedance was chosen throughout this study as this is the fundamental frequency of an action potential and the value commonly used to report impedance in other studies (Ward et al., 2009). Figure 6 shows the 1 kHz impedance trends and the array yield during the implant period for 4 long-term animals (F2, F4, F6: 6 months; F8: 3 months). The figure plots the average array yield (red bars) and average impedance (blue trace) from 16 electrodes on each of the recording days. Impedance values increased progressively to 2–3 times the post-implant values by the 1-week period. In general, impedance values continued to increase until the 3–4 week period with values in the range 1.5–2.5 MΩ after which they decreased. There was also greater variation in the daily impedance values as suggested by the large error bars. The impedance values generally decreased after the 15-day period with occasional fluctuations in the chronic period thereafter. These general impedance magnitude trends with large daily variations were consistent among all animals. Array yield generally was high post-implantation and declined in the days following surgery. In general, an inverse relationship between the daily impedance measurements and the array yield was observed for all our long-term animals.

### Histopathology observations

**Figure 7:**
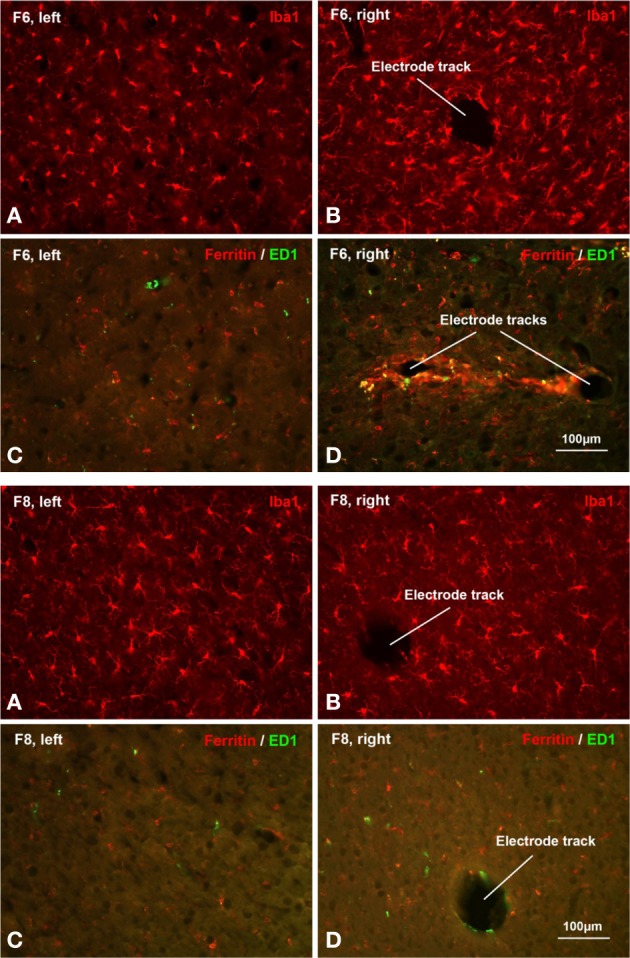
**Histopathology**. Histopathological staining of microglial cells in two chronically implanted animals, F8 (91 days, top panel) and F6 (180 days, bottom panel). In F8, poor electrode array yield was correlated with minimal microglial activation. Comparing Iba1 labeling on the non-implanted contralateral side **(A)** with the implanted side **(B)** it is evident that microglia display a characteristic resting morphology as well as similar density on both sides. There was only modest upregulation of ED1 and ferritin expression in F8, as seen in Table 2 and in panels **(C,D)**. In F6, a moderate-to-good array yield correlated with minimal microglial activation. Comparing panels **(A,B)** microglia appear slightly hypertrophic and somewhat increased in number. Ferritin expression was upregulated modestly and to a similar extent as in F8 (Table 2). Panel **(D)** shows an example of the maximal ferritin upregulation observed around electrode tracks in this animal.

**Figure 8:**
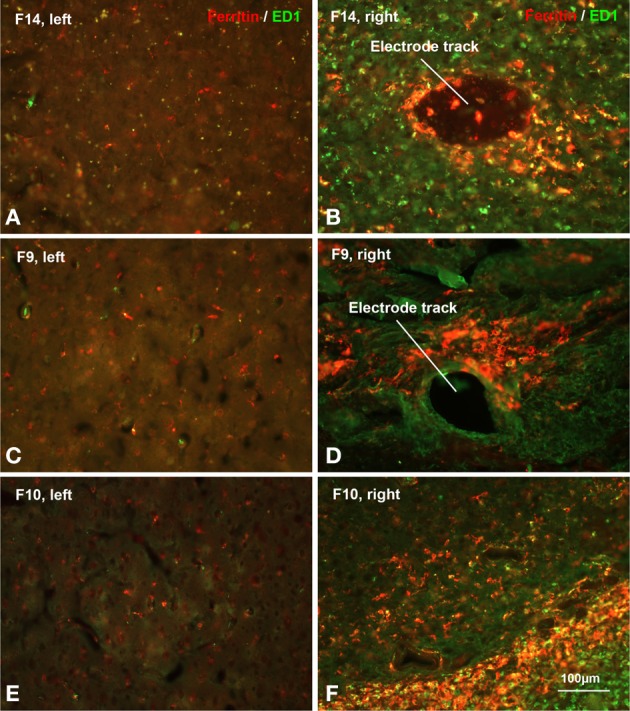
**Hemorrhage**. Increased ferritin expression in animals with poor electrode performance. Shown are micrographs from three different rats all illustrating dramatic upregulation of ferritin (see also Table 2). **(A,B)** F14 is 7 dpi (days post-implantation) and shows a 20-fold increase; **(C,D)** F9 is 71 dpi and shows a 48-fold increase; **(E,F)** F10 is 21 dpi and shows a 91-fold increase in ferritin expression over the control (left) side. The animals also show strong upregulation of both Iba1 and ED1 markers (Table 2). These histopathological findings are indicative of significant tissue damage, hemorrhage, and BBB disruption.

Light microscopic examination of sections stained with microglial markers revealed a rather heterogeneous picture of histopathological changes (Figure 7), but some clear trends were apparent. In general microglial activation, as judged by cell hypertrophy and cell density on Iba1-stained preparations was strongest during early survival times and tended to decrease with increasing survival post-implantation, that is the intensity of neuroinflammation declined over time. Expression of ED1 antigen, indicative of ongoing phagocytic activity, was variable and showed no apparent correlation with post-implant survival times. The ED1 antigen was always present in microglia/macrophages. Expression of ferritin was not related to post-implantation survival times but instead was variable and likely related directly to the extent of vascular damage and hemorrhage (Figure 8). Dense encapsulation of electrodes was not observed in many of the animals and at most involved two cell layers surrounding electrode tracks (Table 2). The extent of encapsulation was not correlated with electrode performance, e.g., F8 (Figure 7; Table 2).

**Table 2:** **Histopathology**.

**Animal groups**	**Rat No**.	**Days post-implant**	**Iba1**		**ED1**		**Ferritin**	
			**Left**	**Right**	**Left**	**Right**	**Left**	**Right**
Short-term implants (≤15 days)	F11	15	2819	10,901	66	826	487	7104
	F12	7	2226	14,248	74	998	745	18,330
	F13	15	4219	14,747	78	687	288	2685
	F14	7	1731	15,941	131	881	248	4974
	F15	6	4761	17,966	19	999	367	5925
Chronic implants (≤6 months)	F9	71	850	16,137	16	253	64	3094
	F10	21	1555	15,149	59	1157	78	7106
	F2	180	3519	4133	594	280	911	2273
	F4	180	2402	3158	260	385	976	2481
	F6	180	1836	2647	125	125	950	2839
	F7	90	4058	4727	35	429	299	4893
	F8	91	1495	2225	24	74	493	1401

Total positive areas of each of the stains for all animals with Pt/Ir array implants. Right refers to the implanted tissue and left refers to the contralateral control tissue.

### Combined abiotic-biotic analysis

**Figure 9:**
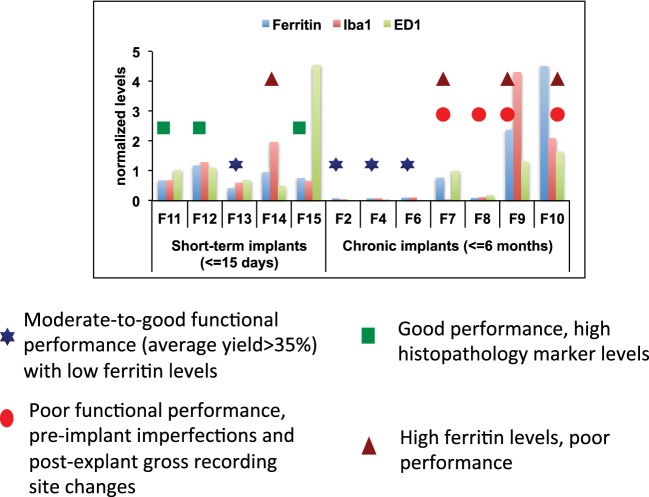
**Combined analysis**. Combined abiotic-biotic analysis to include histological, morphological, and functional performance for all animals to indicate contributing failure modes.

One or more metrics can be used for predicting electrode failure through modeling approaches that would provide us with a reliable estimate of electrode failure. A combined analysis that included histopathological and morphological evaluation combined with functional assessment of these arrays was performed to support our hypothesis of the various abiotic and biotic failure modes that contributes to electrode failure (Figure 9). All histopathological markers (Iba1, ED1, and Ferritin) were normalized across all animals for comparison. We used the following measures to define functional performance: poor (<25% array yield), moderate (25–70% array yield), and good (>70% array yield). All the longest term animals (6-months implant: F2, F4, F6) had moderate to good performance with low histopathology marker levels. Among animals that had good performance, 4 out of 7 animals had very low levels of ferritin expression. The animals (F11, F12, F15) which had higher ferritin levels were very short-term implants. It is likely that ferritin along with other markers were upregulated due to surgical trauma. Even then, the ferritin levels in these animals were much lower than other animals (F7, F9, F10). Therefore, low levels of ferritin was correlated with better functional performance. Electrodes that were poorly manufactured to begin with underwent gross morphological changes overall and had poor functional performance (F7, F8, F9, F10). This supports our hypothesis that insulation delamination/cracking and manufacturing variability leads to reduced impedance over time and degradation in functional performance. There were animals (F11, F12, F15) that had good performance with high histopathology markers which is consistent with our hypothesis that neuroinflammation was not correlated with electrode performance.

## Discussion

We presented in this study an *in vivo* abiotic and biotic characterization approach for Pt/Ir microelectrode arrays in chronic implants. The study combined SEM imaging, impedance spectroscopy, histopathology with electrode functional performance so that better understanding of long-term microelectrode performance can be achieved. Abiotic and biotic measurements were performed over 6-months of implant duration revealing changes that occurred at the electrode-tissue interface affecting functional performance of the microelectrodes. This study was predicated on the hypothesis that failure of this electrode type occurred as a result of both abiotic and biotic factors. Pre-implant SEM imaging showed significant variation between electrodes even within the same array indicating manufacturing imperfections. Post-explant SEM imaging indicated that Pt/Ir electrodes did not suffer from deterioration due to corrosion like tungsten (Prasad et al., 2012), however electrodes exhibited deformation of the recording sites such as bent or cracked tips. In addition, deterioration of insulation material was observed among all electrodes in all animals in the form of insulation delamination and cracking. Generally, electrode impedance reduced chronically in electrodes that had insulation deterioration and poor functional performance. Histopathological evaluation suggested modest elevation of microglial markers for longest-term animals. In contrast, we show evidence in this study that the insulation deterioration, manufacturing, and corrosion were the likely abiotic factors contributing to the electrode failure. In addition to the chronic abiotic and biotic effects, we provide evidence that vascular disruption and concurrent intraparenchymal hemorrhaging during brain implantation is a major biotic factor that likely contributes to electrode failure.

The results of this study indicate a need for precision and consistency of manufacturing of microelectrode arrays, an improvement that will likely play a major role in the long-term performance (Prasad and Sanchez, 2012; Prasad et al., 2012; Streit et al., 2012; Barrese et al., 2013). Of particular interest is the recording site itself and interface between that site and the surrounding insulation. Pre-implant variations such as those depicted in Figure 3 provide a weak or fault site on the microelectrodes that will lead to an accelerated deterioration of the recording surface of individual electrodes. We observed that electrodes with imperfections prior to implantation were more susceptible to morphological changes during the implant period (Figure 5). Manufacturing defects at the recording sites provided fault sites where accelerated deterioration happened resulting in a decreased electrode impedance and poor functional performance (Figure 5). Therefore, to mitigate the variability arising out of the manufacturing process, greater quality control measures should be employed pre-implant to ensure that implanted electrodes are all of good quality. The manufacturer of the FMAs manipulated the amount of deinsulation for each microwire during the laser cutting process to control the impedance. For this study, FMAs were custom-ordered with 100–150 KΩ as the impedance for each microwire. That value of impedance was chosen based upon our experience with tungsten microwires that had the best electrode yield for the impedance range 40–150 KΩ (Prasad and Sanchez, 2012). Pre-implant baseline impedance measurements for these Pt/Ir FMAs were in the range 100–400 KΩ at 1 kHz measured in PBS. While the intent of the deinsulation process was to control impedance, observations from the electrodes used in this study revealed that the process is not as precise as would be desired and there is inconsistency in the manufacturing process (Figure 3). Variance in the starting impedance and manufacturing is compounded by changes in impedance following surgery, which were approximately 2–3 times compared to that measured *in vitro*. The impedance increased progressively over the next 4-week period reaching up to 2–3 MΩ. We observed larger increase in impedance value for these microwires in the first 2–3 weeks as compared to tungsten microwires during the same time period (Williams et al., 2007; Ward et al., 2009; Prasad et al., 2012). While in general, impedance value for tungsten microwires was in the range 30–400 KΩ (Prasad and Sanchez, 2012), the impedance of Pt/Ir microwires were in the order of 1–3 MΩ for most electrodes during the course of chronic implant. This may be attributed to the sharp recording surfaces (smaller geometric surface area) of the Pt/Ir microwires compared to blunt cut recording tips (larger geometric surface area) of tungsten microwires. We and others have reported for tungsten microwires large daily variation in impedance values within an array during the first 3–4 weeks after which the impedance reduced and there were smaller daily variations in the chronic period (Ward et al., 2009; Prasad and Sanchez, 2012; Prasad et al., 2012). In comparison, there was greater day-to-day variability in impedances for Pt/Ir microwires during the entire implant duration as suggested by large error bars (Figure 6). In addition to the abiotic factors affecting electrode impedance, the trends in impedance during the chronic lifetime of the electrodes also suggest the involvement of biotic factors. The cellular changes occurring in the local environment of the electrode tips is a likely contributor of the daily changes in the observed electrode impedance (Williams et al., 2007).

In addition to the electrode insulation interface, our results strongly suggest the bulk insulation itself is an important contributor leading to electrode failure. We have evaluated two of the most commonly used insulation materials in neural electrodes: Polyimide coated tungsten wires (Prasad et al., 2012) and Parylene-C coated Pt/Ir wires (in this study). Insulation delamination and cracking was evident among all wires across all animals for both insulation types (Figure 4B). However, there was a large variation in the extent of the insulation deterioration even between electrodes in the same array. We observed electrodes that had insulation damage pre-implant were more prone to undergo insulation deterioration during the chronic implant (Figure 5). Insulation damage results in leakage resistance and parasitic capacitance (Kane et al., 2013) and continuous decrease in impedance, which leads to an accelerated damage causing decrease in electrode functional performance (Figure 5). These results indicate that electrode delamination and cracking to be a significant failure mode and steps must be taken in order to evaluate better electrode insulation materials in future.

Material differences should also be considered while choosing electrode metals for recording purposes. In previous studies, we have observed that tungsten undergoes rapid corrosion *in vitro* (Patrick et al., 2011) and *in vivo* for implants as early as 1-week following surgery (Prasad et al., 2012). We reported that deterioration of tungsten continued in the chronic phase in the form of recording sites becoming more recessed. Therefore, choice of recording material was one of the contributing abiotic failure modes for tungsten electrodes. However, we observed in this study that Pt/Ir provides better recording surfaces and corrosion was not as big of an issue as with tungsten. Figure 4A shows various Pt/Ir electrodes both pre-implant and post-explant for varying implant durations (7 days–6 months). We can observe that corrosion was not a significant problem. We did not observe recessed recording sites like tungsten in any of the electrodes and across animals. Even for electrodes, which underwent large morphological changes at the electrode recording sites (Figure 4B), electrodes were not as much affected by corrosion. Therefore, corrosion does not appear to be a significant contributing factor to electrode failure for Pt/Ir electrodes.

One of the experimental design factors that enabled deeper insight to electrode failure is that we coupled electrode array functional performance with impedance spectroscopy to study if there was a functional relationship between them. Electrode array functional performance was quantified by array yield which was defined as the percentage of electrodes in an array that were able to isolate single units. Higher array yield are desirable for a neuroprosthetic application. In general for these floating arrays, 1 kHz impedance magnitude trends were similar for long-term animals where impedance increased progressively in the first 2–3 weeks and then decreased in the subsequent weeks with occasional increases and decreases in the chronic period (Figure 6). We observed that array yield generally declined during this initial 2–3 week period following implant attributed as a result of surgical trauma in other studies (Biran et al., 2005). Poor array yield (<25% yield) was observed when impedance was high (>1.5 MΩ) and yield increased when impedance was low during the late chronic period (>12 weeks) for long-term animals (Figure 6). The daily variations observed in average impedance magnitude corresponded with moderate (25–70% yield) to poor (<25% yield) functional yield from these Pt/Ir MEAs among all animals (Table 1).

There is a cascade of biological reactions that occur following an electrode implant surgery that in brief includes activation and recruitment of astrocytes, microglia, and macrophages at the implant site in order to isolate the implanted device and repair the damaged tissue (Polikov et al., 2005; Thelin et al., 2011; Kozai et al., 2012b). Several studies have pointed out the time course of such biological reactions occurring at the tissue-electrode interface that begin as early as right after insertion and continue as long as the implant is in the neural tissue (Szarowski et al., 2003; Freire et al., 2011; Prasad et al., 2012). Of course, the most obvious and inevitable biological consequence of intracerebral electrode insertion, or for that matter any type of traumatic brain injury, is a neuroinflammatory response. Neuroinflammation is mediated primarily by activated microglial cells which is why we have focused our attention on these cells in this and prior studies (Prasad et al., 2011, 2012; Streit et al., 2012). Our findings with regard to activated microglia were as expected in that electrode implantation elicited widespread microglial activation at the implantation site, which in most cases became somewhat attenuated over time. However, we also studied microglial degeneration, evident as cytoplasmic fragmentation, which we believe is a direct consequence of oxidative stress brought about by influx of free iron through intracerebral bleeding. In contrast to our prior study with tungsten microwires where microglial degeneration was observed only during early survival times, the current study shows that microglial degeneration can also occur during relatively late time points (Figure 8), especially in animals where there was significant intracerebral bleeding thus supporting our belief that microglial cytoplasmic fragmentation occurs as a direct consequence of iron-mediated oxidative stress.

In conclusion, the results of our study serve to further illuminate the problem of electrode failure by focusing attention on two major failure modes, one abiotic and the other one biotic. Future efforts geared toward prolonging the functional lifetime of electrodes should thus be focused on further improving electrode manufacturing practices and on innovative ways of minimizing intracerebral bleeding and associated oxidative stress.

### Conflict of interest statement

The authors declare that the research was conducted in the absence of any commercial or financial relationships that could be construed as a potential conflict of interest.
